# The case for mandatory – not voluntary – front-of-package nutrition labels

**DOI:** 10.2471/BLT.24.292537

**Published:** 2024-09-11

**Authors:** Lindsey Smith Taillie, Ana Clara Duran

**Affiliations:** a Gillings School of Global Public Health, University of North Carolina at Chapel Hill, Chapel Hill, United States of America.; b Center for Food Studies and Research (NEPA), University of Campinas, Av. Albert Einstein, 291, 13083-852 Campinas, Brazil.

**Fig. 1:**
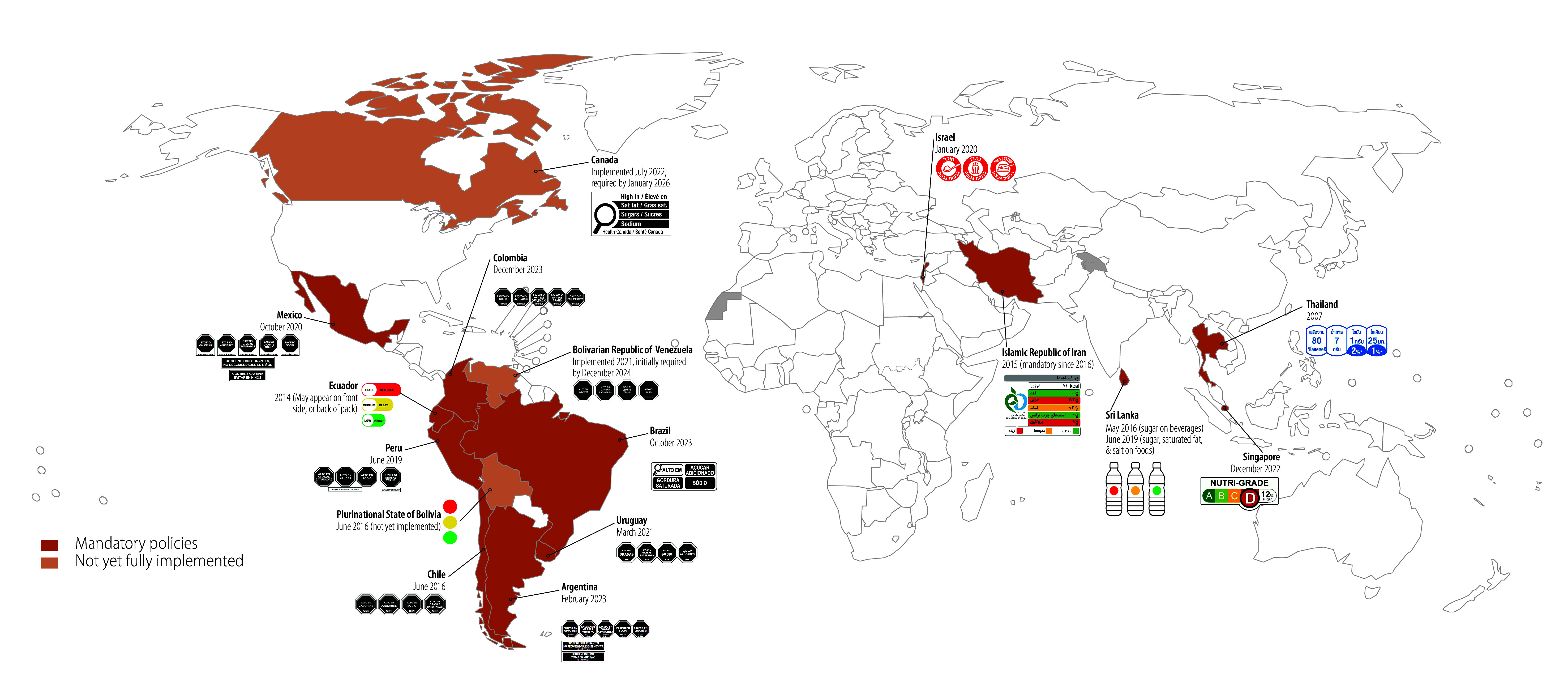
Countries with mandatory front-of-package nutrition labelling policies

In recent decades, policy-makers have taken policy actions to improve dietary quality and prevent further increases in obesity, type 2 diabetes and other noncommunicable diseases, including policies that require mandatory placement of a nutrition label on the front of food and beverage packages, with 15 countries currently requiring such a label (Fig. 1). Thirty-six other countries have voluntary front-of-package nutrition label policies that recommend but do not mandate companies to label their products. These policies aim to inform consumers about the nutritional content of prepackaged foods and beverages, and indirectly encourage manufacturers to reformulate their products to reduce the content of targeted nutrients or ingredients. 

However, front-of-package nutrition label policies have varying degrees of effectiveness, based on whether they are mandatory or voluntary. Mandatory front-of-package nutrition label policies outperform voluntary ones in driving food reformulation, informing consumers and promoting healthier choices. Real-world evaluations show that when these policies are mandatory, particularly when the mandated label is a nutrient warning, manufacturers improve the nutritional content of the food supply, and consumers reduce purchases and intakes of products high in nutrients of concern.^1^^,^^2^ In contrast, little data confirm dietary improvements when implementation of the policies is voluntary. For example, an evaluation of the United Kingdom of Great Britain and Northern Ireland’s voluntary traffic-light labelling system found no effect on the healthiness of consumer food purchases.^3^ A recent review of the voluntary Nutri-score label implemented in seven European countries (Belgium, France, Germany, Luxembourg, the Kingdom of the Netherlands, Spain and Switzerland) found that most positive studies were conducted by Nutri-score’s developers, while most independent studies reported negative results.^4^

Additionally, the voluntary – and not mandatory – application of Australia’s Health Star Ratings is partly behind the little to no changes observed in consumer purchasing behaviour.^5^ A study^6^ shows that the uptake of Australia’s voluntary Health Star Rating label was much lower than the uptake of the mandatory country-of-origin label (39% or 8637/22 147 products eligible to receive the Health Star Rating label, versus 93% or 24 039/25 848 products eligible for a mandatory country-of-origin label in 2023). Voluntary labelling policies also allow for inconsistent application, enabling companies to selectively label healthier products while omitting labels on less healthy ones. In Australia, products displaying the Health Star Rating had a higher mean rating than those without.^7^ This selective labelling may lead to consumer misinformation, as unhealthier products are less likely to display labels that would help consumers identify them as unhealthy. In contrast, mandatory policies apply across all foods and beverages, with data from countries with mandatory nutrient warnings showing high compliance. For example, recent data from Chile show that 93% (6168/6589) of products high in unhealthy nutrients identified in the sample that were required to carry a warning label do display the label.^8^

**Fig. 2:**
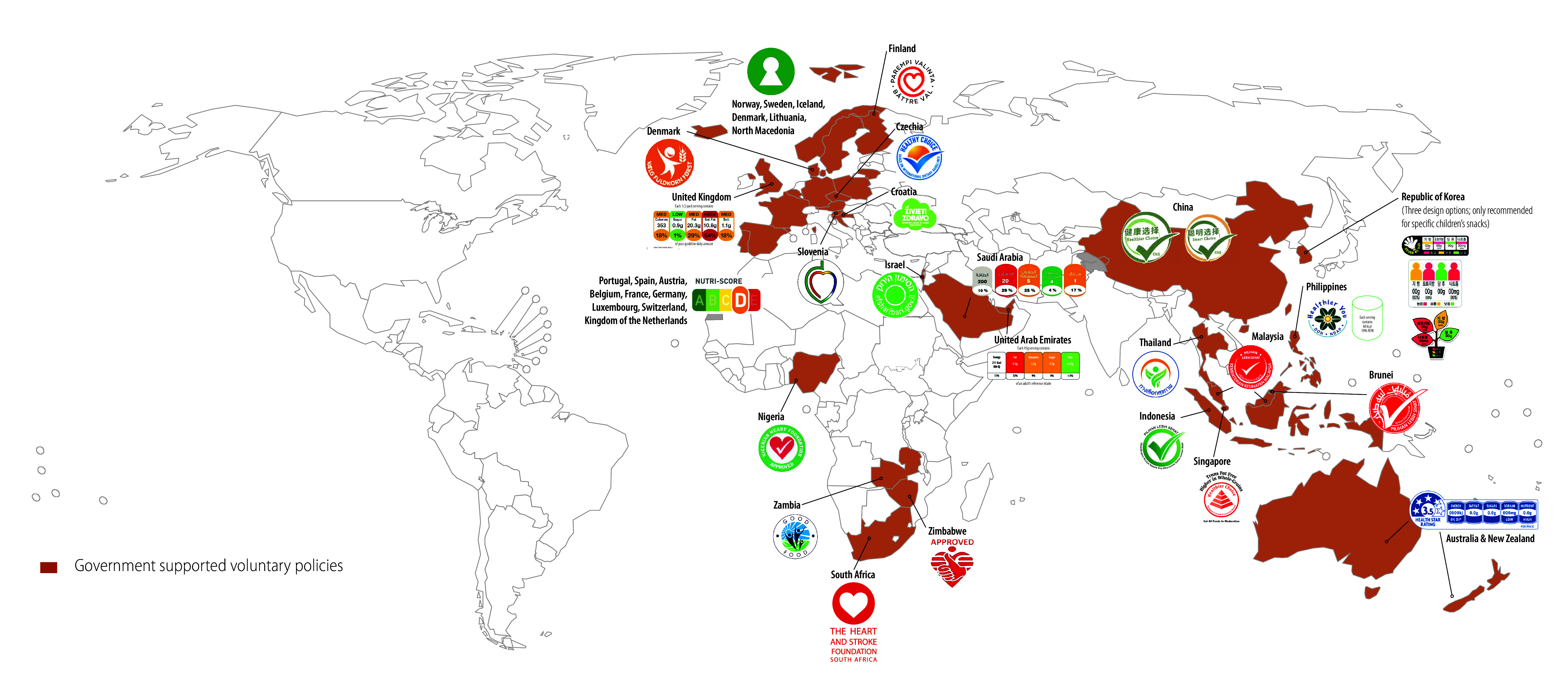
Countries with voluntary front-of-package nutrition labelling policies

Another reason why voluntary policies tend to be less effective is their design. Most mandatory policies use a nutrient warning design, which signals that a product is high in a nutrient of concern. Ample data show that nutrient warnings grab attention, are easy to understand, improve people’s ability to identify unhealthy products and reduce selection of unhealthy products.^9^ In contrast, voluntary schemes vary greatly, ranging from colour-coded traffic light labels, numeric daily guideline allowance labels, summary measures and healthy icons or checks (Fig. 2).

Mandatory nutrient warnings typically outperform voluntary labels like the traffic light and daily guideline allowance labels, as they offer a clear, binary signal that is easier for consumers to quickly understand, unlike systems requiring the consideration of multiple, sometimes conflicting, pieces of information.^10^ For instance, a product can be low in sodium but high in added sugars, which can lead to confusion and misperceptions about a product’s healthfulness. No country to date has a voluntary nutrient warning label system, suggesting that mandatory governmental-led regulation may be the only strategy for implementing this labelling scheme.

Finally, mandatory labelling policies typically employ more robust, evidence-based and stricter nutrient profile models, which are vital for accurately identifying unhealthy products in front-of-package labelling systems. Nutrient profile models employed by many countries with mandatory labelling policies, such as those adopted in Chile and Mexico, better identify ultra-processed foods because they target products high in sugar, sodium, saturated fat and more recently, non-sugar sweeteners. For instance, Mexico adopted the innovative policy of adding non-sugar sweeteners to mandatory labelling systems, which was intended to prevent companies substituting non-sugar sweeteners for added sugars, as occurred in Chile.^11^ This addition to mandatory labelling policies is important, since it captures an additional subset of ultra-processed foods – that is, those that may not be high in added sugar but still contain this class of additives. 

In contrast, three out of four products launched in the Australia food supply between 2014 and 2017 that displayed the voluntary Health Star Rating were classified as ultra-processed.^12^ Moreover, voluntary schemes like Nutri-Score and Health Star Rating use summary algorithms that award points for positive nutrients and ingredients, despite the presence of harmful ingredients and nutrients, which can lead to positive ratings even for ultra-processed foods. This approach does not reflect the existing scientific evidence, which does not indicate that the inclusion of ingredients such as dried fruit or fibre powder offsets the harms of added sugars or sodium. 

Mandatory front-of-package nutrition labels are crucial for guiding consumers towards healthier food choices, which are in turn influenced by several factors, including availability, price and marketing. An optimal policy would be combined with measures that address these influences – for instance, by prohibiting nutrition and health claims and child-directed marketing techniques on products that carry warning labels. In addition, because most front-of-package nutrition labelling schemes only apply to prepackaged foods, unpackaged fresh and minimally processed foods like fruits, vegetables, bulk grains and bulk cereals are not subject to front-of-package nutrition labelling policies. Additional policies are needed to ensure access and affordability of healthy foods and incentivize their consumption. These policies could include securing, promoting and expanding fresh produce markets; financial subsidies or assistance programmes to increase affordability of healthy foods; and taxes on ultra-processed foods that could generate revenue for additional healthy food promotion programmes. In addition to these measures, policy-makers can use front-of-package nutrition labels as groundwork for broader regulations such as bans of ultra-processed food in schools and government food procurement, taxes and marketing restrictions; as well as more upstream issues such as industry interference, social, racial and gender inequalities, and climate change.
