# Corrigendum

**DOI:** 10.2471/BLT.24.111124

**Published:** 2024-11-01

In: Smith Taillie L, Duran AC. The case for mandatory – not voluntary – front-of-package nutrition labels. Bull World Health Organ. 2024 Oct 1; 102 (10):765–8, 

on page 765, second paragraph, sixth and seventh sentences should read as follows:

A recent review of the voluntary Nutri-score label implemented in seven European countries (Belgium, France, Germany, Luxembourg, the Kingdom of the Netherlands, Spain and Switzerland) found that most positive studies were conducted by Nutri-score’s developers, while most independent studies reported negative results.^4^ However, this assessment should be interpreted with caution, since the review authors themselves may have had a conflict of interest due to industry funding.

